# Variation in carbon sequestration in response to water limitation in a diverse panel of switchgrass genotypes

**DOI:** 10.1002/jeq2.70118

**Published:** 2025-12-11

**Authors:** Anita Giabardo, Kishan Mahmud, Shiva Makaju, Gary Hawkins, Ali Missaoui

**Affiliations:** ^1^ Department of Crop and Soil Sciences University of Georgia Athens Georgia USA; ^2^ Institute of Plant Breeding Genetics and Genomics University of Georgia Athens Georgia USA; ^3^ Current address: Purple Cow Organics Waunakee Wisconsin USA

## Abstract

Switchgrass (*Panicum virgatum* L.) has been identified as a “model” herbaceous species for bioenergy production by the United States Department of Energy. Switchgrass can provide several ecosystem services, including biodiversity support, soil erosion control, runoff filtering, and reclamation of marginal land. In addition to the reduction in greenhouse gas emissions from switchgrass‐derived biofuel, soil carbon sequestration is of particular importance. The objective of this study was to evaluate the variability in soil carbon sequestration, particularly in response to water limitation, and to investigate the relationship between soil carbon sequestration and switchgrass yield. For this purpose, dry aboveground biomass yield and soil reactive carbon—specifically, permanganate oxidizable carbon (POXC)—content at three depths (0–15, 15–30, and 30–60 cm) were measured for 150 different switchgrass genotypes for three consecutive years. We found that drought significantly reduced yield compared to control plots, reduced the amount of soil POXC, and that POXC decreased with soil depth. A positive correlation (*r* = 0.27, *p* < 0.05) between POXC and yield was observed in the drought‐stressed plots. This study provides insight into the impact of switchgrass on soil POXC over time and at different depths, offering a framework for future evaluation of root‐related traits in switchgrass, particularly in relation to drought stress.

## Core Ideas

- The relationship between water limitation, yield, and readily accessible organic matter in switchgrass was investigated.
- Readily accessible organic matter content was measured as permanganate oxidizable carbon (POXC) over multiple growing seasons.
- Water limitation decreased soil POXC content and revealed a mildly positive correlation between POXC and yield.

## Plain Language Summary

Switchgrass has been designated as a model herbaceous species for biofuel production by the United States Department of Energy. Switchgrass can provide several ecosystem services, including biodiversity support, soil erosion control, runoff filtering, and reclamation of marginal land. In addition to the reduction in greenhouse gas emissions from switchgrass‐derived biofuel, soil carbon sequestration is of particular importance. The objective of this study was to study the relationship between soil carbon sequestration and switchgrass yield under simulated drought conditions. For this purpose, switchgrass yield and permanganate oxidizable carbon (POXC)—a measure of soil reactive carbon—were measured for 150 different switchgrass genotypes for three consecutive years. We found that drought reduced the amount of soil POXC, and we observed a mildly positive correlation between POXC and yield under drought conditions.

**Abbreviations**
%BS: percent base saturation
%OM: percent organic matter
%TOC: percent total organic carbon
CEC: cation exchange capacity
CV: covered
POXC: permanganate oxidizable carbon
SOC: soil organic carbon
UC: uncovered

## 1 INTRODUCTION

Switchgrass (*Panicum virgatum* L.) is a monocot C_4_ perennial grass native to North America and a member of the *Poaceae* family, subfamily *Panicoideae*. It is found throughout most of the United States (U. S. Department of Energy, 2011) and is recognized as a “multipurpose crop species” (Parrish & Fike, 2005). Switchgrass provides several ecosystem services, including biodiversity enhancement (Werling et al., 2014), soil erosion control (Wang et al., 2020), vegetative filtering of surface runoff, and reclamation of marginal land (Munshower, 1994). Switchgrass has been identified as a “model” herbaceous species for bioenergy production by the U.S. Department of Energy (Mclaughlin & Kszos, 2005). Thanks to its outstanding capabilities for biomass accumulation and thus carbon storage, switchgrass cropping can contribute to soil health improvement and greenhouse gas sequestration through the uptake of atmospheric CO_2_ (Liebig et al., 2005).

Switchgrass is typically harvested annually, with the optimum harvest time varying by state in the United States (Makaju et al., 2013). Most of the leaves remain intact at harvest; therefore, any contribution to soil C sequestration is attributable to root‐soil interface dynamics and belowground C storage. However, the rate at which soil carbon content increases and the depths at which carbon sequestration occurs can vary. The developed rootstock of switchgrass allows it to store soil organic carbon (SOC) not only at the soil surface but also at deeper depths, where carbon is generally less susceptible to mineralization and loss (Liebig et al., 2005). SOC supplies key energy to the soil food web and biological activities (Lal, 2016). For the long‐term sustainability of bioenergy crops such as switchgrass, soil C sequestration may mean improved soil nutrient cycling and enhancement of other soil functions (Follett et al., 2012). Lemus and Lal (2005) and Robertson et al. (2008) reported the importance of carbon sequestration in relation to a number of ecosystem services, such as degraded soil restoration, soil erosion control, and overall soil quality enhancement. The authors estimate that bioenergy crops have the potential to sequester 20% of the total US annual emissions, considering biomass yield, dedicated land, C sequestration potential, and conversion efficiency. Given the C trading market growth, they additionally suggest that the sequestered carbon could become a substantial new form of income for farmers.

The wide variation in aboveground biomass yield and the considerable belowground rootstock of switchgrass suggest that such phenotypic variability could be reflected in belowground rootstock architecture. These differences may influence variation in root decomposition rates (de Graaf et al., 2013) and, consequently, SOC turnover. In addition, there is variation in the degree of drought tolerance among different switchgrass genotypes (Liu et al., 2015). However, our understanding of the interplay between drought resistance, soil C sequestration, and genotype is limited.

The fraction of carbon measured by chemical oxidation with potassium permanganate, known as permanganate oxidizable carbon (POXC) (Culman et al., 2012; Jagadamma et al., 2019; Liptzin et al., 2022; Weil et al., 2003), reflects the readily accessible portion of soil organic matter that can be easily catabolized by microbes. POXC is also an indicator of rapid changes in SOC due to changes in agronomic management (Culman et al., 2012; Hurisso et al., 2016; Weil et al., 2003). Using POXC as a measure of soil active C, the objectives of this research were to (a) measure active soil C under drought treatment within a diverse panel of 150 switchgrass genotypes at three different soil depths (0–15, 15–30, and 30–60 cm) and (b) investigate the relationship between POXC and yield, also in relation to drought conditions.

## 2 MATERIALS AND METHODS

### 2.1 Experimental site and design

Four hundred five different genotypes were tested under two treatments (1) control (or “uncovered” [UC]) and (2) water limitation (or “covered” [CV]). The experimental plots under study are located within the University of Georgia's Gibbs Farm (Tifton, Tift County, GA; 31.4415007° N, 83.5799678° W, 116 m elevation). The soil is classified as fine‐loamy, kaolinitic, thermic, Plinthic Kandudults (Soil Survey Staff, 2020). Climatic data of Tift County, including monthly minimum temperature, maximum temperature, and average rainfall, are provided in Table S1 (University of Georgia Weather Network, 2023). The average soil pH in 2018 was 6.30 in the UC plots and 6.70 in the CV plots. Tile drains were placed underneath the CV plot at about 1.2 m depth to prevent the water table from rising. PVC well screen pipes were placed at the four corners of the rain exclusion shelter (CV plots) to allow for monitoring of the depth of the water table. Soil volumetric water content and temperature were monitored by sensors placed inside the shelter (AQUA TRAC, AgSense) and validated throughout the year by means of a field scout digital moisture sensor (TDR 350 Soil Moisture Meter, Turf‐Tec International).

**FIGURE 1:**
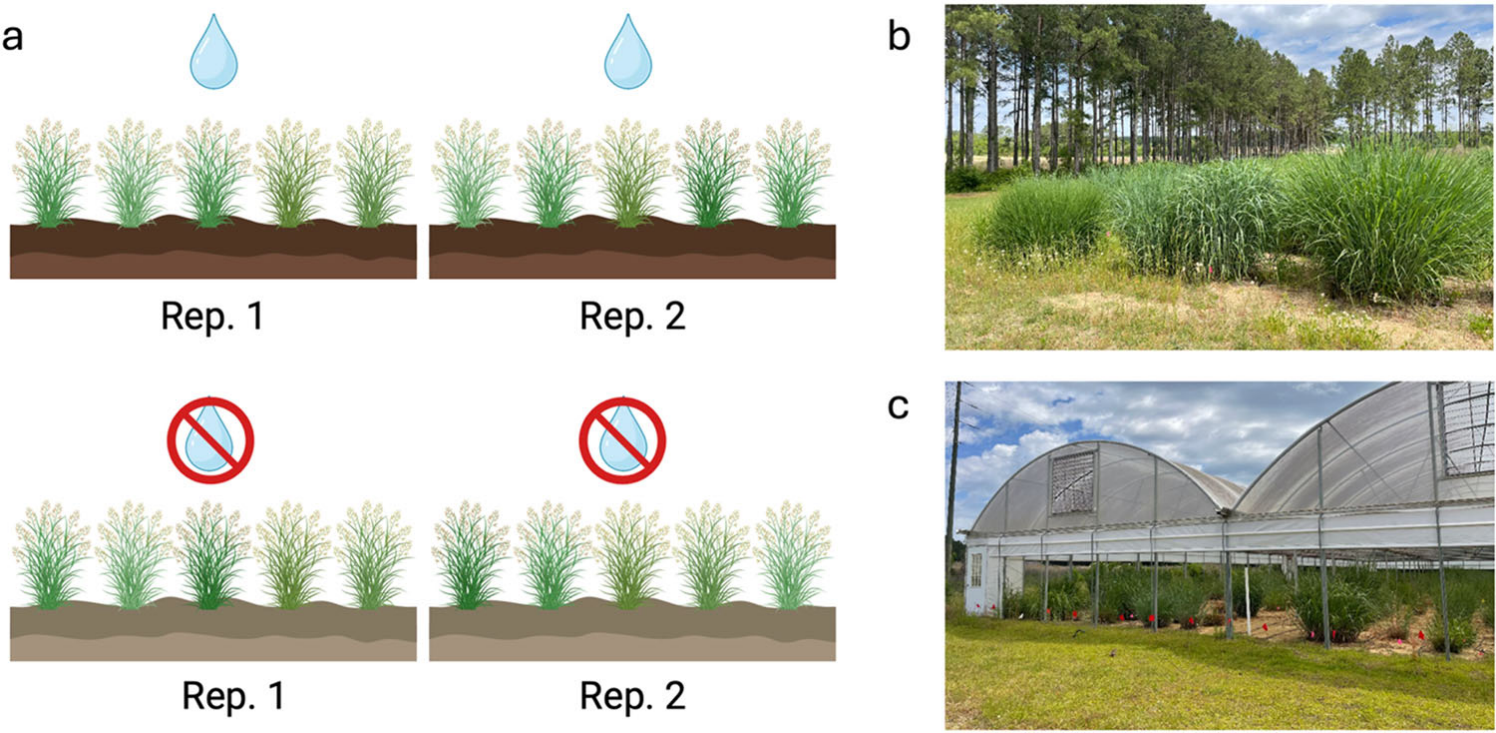
Schematic representation of the experimental design (a). Pictures of the UC—“uncovered” plot (b) and the CV—“covered” (c) plot with rain exclusion shelter. Created in BioRender (https://BioRender.com/u72p688) (Giabardo, 2025).

The drought treatment was maintained using an automated rain exclusion shelter (54.8 m long × 26.1 m wide × 2.3 m tall, with a maximum height of 4.5 m reached at the middle of each module—see Figure 1c) that closes all sides automatically when it senses rain, which prevents rainwater from reaching the plants. Irrigation was only applied during early spring for fertilization. After that, no further irrigation was applied, simulating realistic drought conditions without causing severe plant death. Figure 1 shows a schematic representation of the experimental layout. In the UC plot, plants are exposed to rainwater (Figure 1) and supplemented with drip irrigation to maintain the moisture level near field capacity. For each of the two treatments, three replicates were set up, for a total of around 2400 individual switchgrass plants involved, arranged in a randomized complete block design.

Lovell et al. (2021) developed a diversity panel of 732 switchgrass genotypes at the University of Texas at Austin using seeds, rhizomes, and clonal propagules collected from natural and common garden sources across the United States between 2010 and 2018. From this panel, we received 405 diverse clones and split the tillers to establish two replications in each of the UC and CV treatments in August 2018. Additional tiller clones were propagated in the greenhouse, and the third replication was established in April 2019. Considering the labor and time requirement, this study focused on a subset of 150 genotypes in the first two replicates.

**Core Ideas**

- The relationship between water limitation, yield, and readily accessible organic matter in switchgrass was investigated.
- Readily accessible organic matter content was measured as permanganate oxidizable carbon (POXC) over multiple growing seasons.
- Water limitation decreased soil POXC content and revealed a mildly positive correlation between POXC and yield.

These 150 genotypes were selected based on phenotypic diversity (yield and associated morphological traits) in order to avoid selecting only high‐yielding genotypes. Of the 150 switchgrass genotypes included, 94 are classified as lowland, 40 as coastal, six as upland, and 10 as unknown (Lovell et al., 2021). The collection sites of the germplasm are located in 16 different U.S. states (including Arkansas, Florida, Georgia, Iowa, Kansas, Louisiana, Michigan, Mississippi, Missouri, Nebraska, New Mexico, North Carolina, Oklahoma, South Carolina, Texas, and West Virginia) and Mexico, spanning from 26.86972° to 46.38829° N latitude and 70.7594° to 103.609° W longitude (Lovell et al., 2021). At the time of this research study, the genotypes did not have any information on their performance under drought stress. Both CV and UC trials were established in spring 2018, and within each replicate, the plants were spaced 90 cm apart from each other and arranged into rows and ranges. The covered replicates consisted of seven rows with 58 ranges (Figure S1), while the uncovered included 14 rows with 29 ranges (Figure S2).

### 2.2 Site management history

With regard to previous management, the field where the covered trial was established was cropped with cotton (*Gossypium hirsutum*) and peanut (*Arachis hypogaea*) in a rotation from 2000 to 2008, switchgrass from 2009 to 2011, and again cotton and peanut from 2012 to 2017. Uncovered plots were established on long‐term grasslands, where a mixture of Bahiagrass (*Paspalum notatum*) and Bermudagrass (*Cynodon dactylon*) had been cultivated until 2018. Annually, dead plants were replaced with switchgrass genotype AP13 (Alamo) to prevent neighboring plants from gaining any competitive spatial advantage.

### 2.3 Soil sampling

**FIGURE 2:**
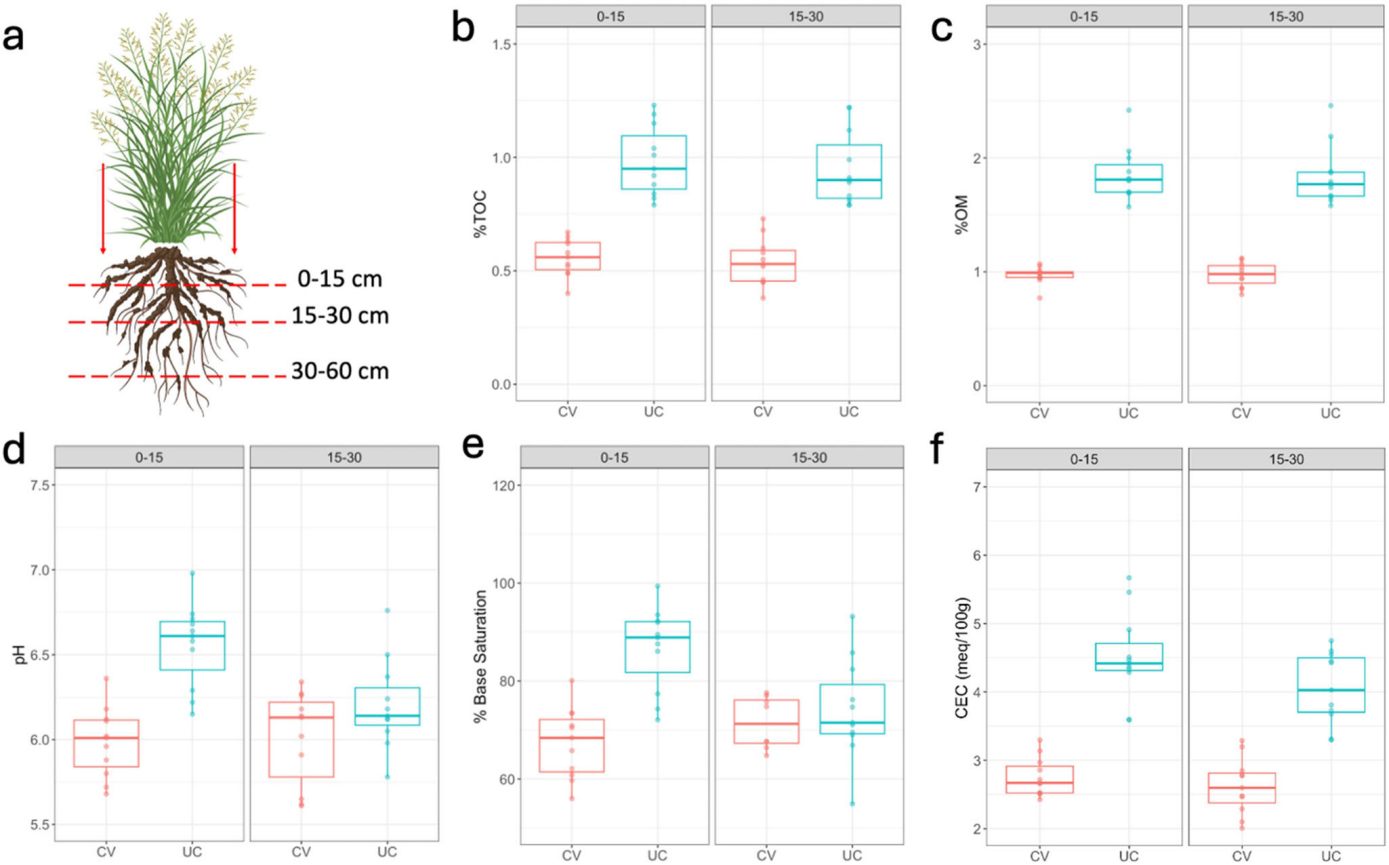
(a) Schematic representation of soil sampling strategy. Two holes are drilled beside the plant, and the soil from the same depth is mixed together in a sample. Soil is sampled at three different depths. Created in BioRender (https://BioRender.com/bhhlna5) (Giabardo, 2025). (b) Percent total soil organic carbon (%TOC) by treatment and depth. (c) Percent soil organic matter (%OM) by treatment and depth. Organic matter was determined using the “loss on ignition” (LOI) method. (d) Soil pH by treatment and depth. (e) Percent base saturation (%) by treatment and depth. (f) Cation exchange capacity (meq/100 g) by treatment and depth. Samples represented in (b), (c), (e), and (f) were collected in April 2023.

In 2020, every plant located within the second replicate of each treatment was subjected to soil sampling at 0‐ to 15‐cm depth, using a push auger with a 2.5 cm diameter. Each sample is a composite of two sub‐samples per plant, taken within 5 cm from the plant base (Figure 2a; Figure S3). The soil samples were collected, stored in paper bags, and air‐dried for 24 h to minimize reactivity without affecting organic carbon content (Tan, 2005), followed by sieving through a 2‐mm sieve.

In January 2021 and March 2022, soil samples were collected from the same 150 selected genotypes. The soils surrounding the corresponding plants were sampled with a drill at three depths (0–15, 15–30, and 30–60 cm). The drill bit (2 cm diameter) was marked at the desired depths to ensure consistent sampling depth. Additional soil sampling was performed in April 2023 on a subset of 10 genotypes to check for differences in soil characteristics after 3 years of switchgrass cultivation and drought treatment. Sampling was performed at 0‐ to 15‐cm and 15‐ to 30‐cm depths.

### 2.4 Biomass harvest

Aboveground biomass of each individual plant was harvested each year, in December. Plants in the CV plots were cut to a height of about 10 cm from the ground using a hand sickle or chainsaw, while the plants in the UC plots were cut to the same height using Wintersteiger Cibus Harvester (Wintersteiger AG). A fresh biomass sample from each plant was collected in a brown paper bag, weighed to record the fresh sample biomass weight, and then dried in a forced air drier at 55°C for at least 72 h. After drying, the sample was weighed again to record the dry sample biomass weight. The dry matter content, calculated from the fresh and dry sample biomass weights, was used to determine the dry biomass yield of the individual plants.

### 2.5 Soil analysis

Soil active carbon, or POXC, was measured following the POXC protocol described by Weil et al. (2003) with minor modifications (Culman et al., 2012). For POXC content, absorbance was measured at a wavelength of 550 nm with a Tecan Infinite M200 microplate reader (Tecan Group Ltd.). A standard curve was constructed using the molarity and the absorbance of the standard solutions each time the spectrophotometric reading was performed. POXC was calculated using the following equation (Weil et al., 2003):

$$\begin{array}{ccc} \mathrm{POXC}\;\left(\mathrm{mg}\;kg^{-1}\right) & = & \left[0.02\frac{\mathrm{mol}}{L}-\left(a+b\times \mathrm{Abs}\right)\right]\\ & & \times \;\left(9000\;\mathrm{mg}\;\frac{C}{\mathrm{mol}}\right)\times \left(\frac{0.02\;L}{\mathrm{Wt}}\right) \end{array}$$

where 0.02 mol/L is the initial solution concentration, *a* is the intercept of the standard curve, *b* is the slope of the standard curve, Abs is the absorbance of unknown sample, 9000 mg C/mol is the milligrams of carbon oxidized by 1 mole of ${\mathrm{MnO}}_{4}^{-}$ changing from Mn^7+^ to Mn^2+^, 0.02 L is the volume of stock solution reacted, and Wt is the weight of air‐dried soil sample expressed in kg.

Additional analysis on a subset of 10 genotypes included soil pH, percent base saturation (%BS), cation exchange capacity (CEC), percent organic matter (%OM), and percent total organic carbon (%TOC). The samples were processed according to the protocols followed by the University of Georgia's Agricultural and Environmental Service Laboratories (2023).

### 2.6 Statistical analysis

The data were analyzed using linear models and linear mixed models in R software (R Core Team, 2018). The models included the fixed effect of treatment, depth (when more than one depth was considered), and year, as well as the random effect of block (or replication) when there was more than one. The models were computed using the *lmer()* function from the lme4 R package (Bates et al., 2015). Post hoc pairwise comparisons were conducted using the *emmeans()* function in the emmeans R package (Lenth, 2023). Analysis of variances (ANOVAs) were carried out using the *Anova()* function in the *car* R package (Fox & Weisberg, 2019).

Pearson's correlation coefficient (*r*) and Spearman's rank correlation coefficient (*ρ*) were calculated to examine the relationship between yield and POXC. Pearson's coefficient measures the strength of a linear association between two continuous variables, assuming normal distribution of data. In contrast, Spearman's coefficient assesses a monotonic relationship based on ranked data and is less sensitive to non‐normal distributions or outliers. The combination of these two coefficients provides a more robust evaluation of the association between aboveground biomass yield and POXC.

## 3 RESULTS

### 3.1 Soil characteristics

The UC plot had an overall higher %TOC (Figure 1b), %OM (Figure 1c), pH (Figure 1d), %BS (Figure 1e), and %CEC (Figure 1f) at both sampling depths 0–15 and 15–30 cm. Post hoc pairwise comparisons are reported in Table 1.

**TABLE 1:** Post hoc pairwise comparisons between covered (CV) and uncovered (UC) plots at different depths for selected soil characteristics.

Soil characteristic	Depth (cm)	Estimate (contrast: CV vs. UC)	SE	*t* ratio	*p*‐value
CEC	0–15	−1.76	0.207	−8.489	**<0.0001**
	15–30	−1.44	0.207	−6.983	**<0.0001**
TOC	0–15	−0.425	0.0557	−7.644	**<0.0001**
	15–30	−0.413	0.0557	−7.416	**<0.0001**
BS	0–15	−19.26	4	−4.811	**<0.0001**
	15–30	−8.52	4	−2.127	**0.0396**
pH	0–15	−0.572	0.106	−5.380	**<0.0001**
	15–30	−0.192	0.106	−1.805	**0.0786**
OM	0–15	−0.890	0.0804	−11.068	**<0.0001**
	15–30	−0.865	0.0804	−10.762	**<0.0001**

*Note*: Comparisons were performed using the emmeans() function in the emmeans package in R software. *p*‐values were adjusted using the Tukey method. *p*‐values in bold are considered statistically significant.

Abbreviations: BS, base saturation; CEC, cation exchange capacity; OM, organic matter; SE, standard error; TOC, total organic carbon.

**FIGURE 3:**
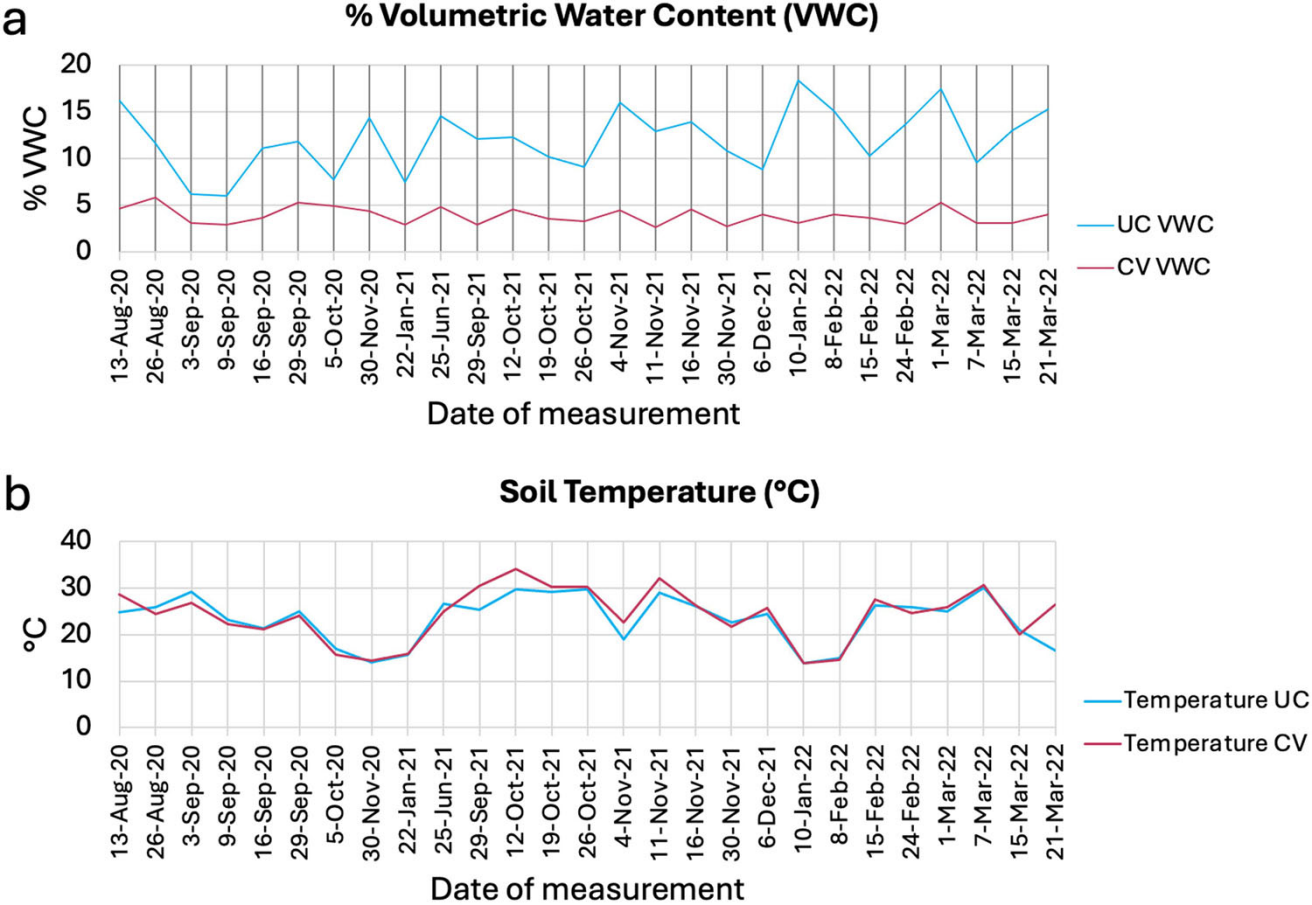
Soil volumetric water content (VWC) (a) and soil temperature (°C) (b) in control (uncovered [UC]) and drought‐stressed (covered [CV]) plots.

Soil temperature and volumetric water content were measured from 2020 through 2022. Volumetric water content data are shown in Figure 3a. Volumetric water content was consistently higher under the UC treatment compared to the CV treatment. Figure 3b shows soil temperature data. Temperatures recorded in the UC and CV plots were similar.

### 3.2 POXC at baseline

The baseline data collected in the year 2020 consisted of soil sampling, sample analyses, and statistical analyses. The model included only the fixed effect of treatment. The average baseline POXC content was 314.15 mg kg^−1^ in the CV plot and 342.58 mg kg^−1^ in the UC plot (Figure S1). The ANOVA revealed no statistically significant difference between the two plots at baseline (*p* = 0.16) (Figure S4).

### 3.3 Effects of drought on POXC and yield across soil depths and years

**FIGURE 4:**
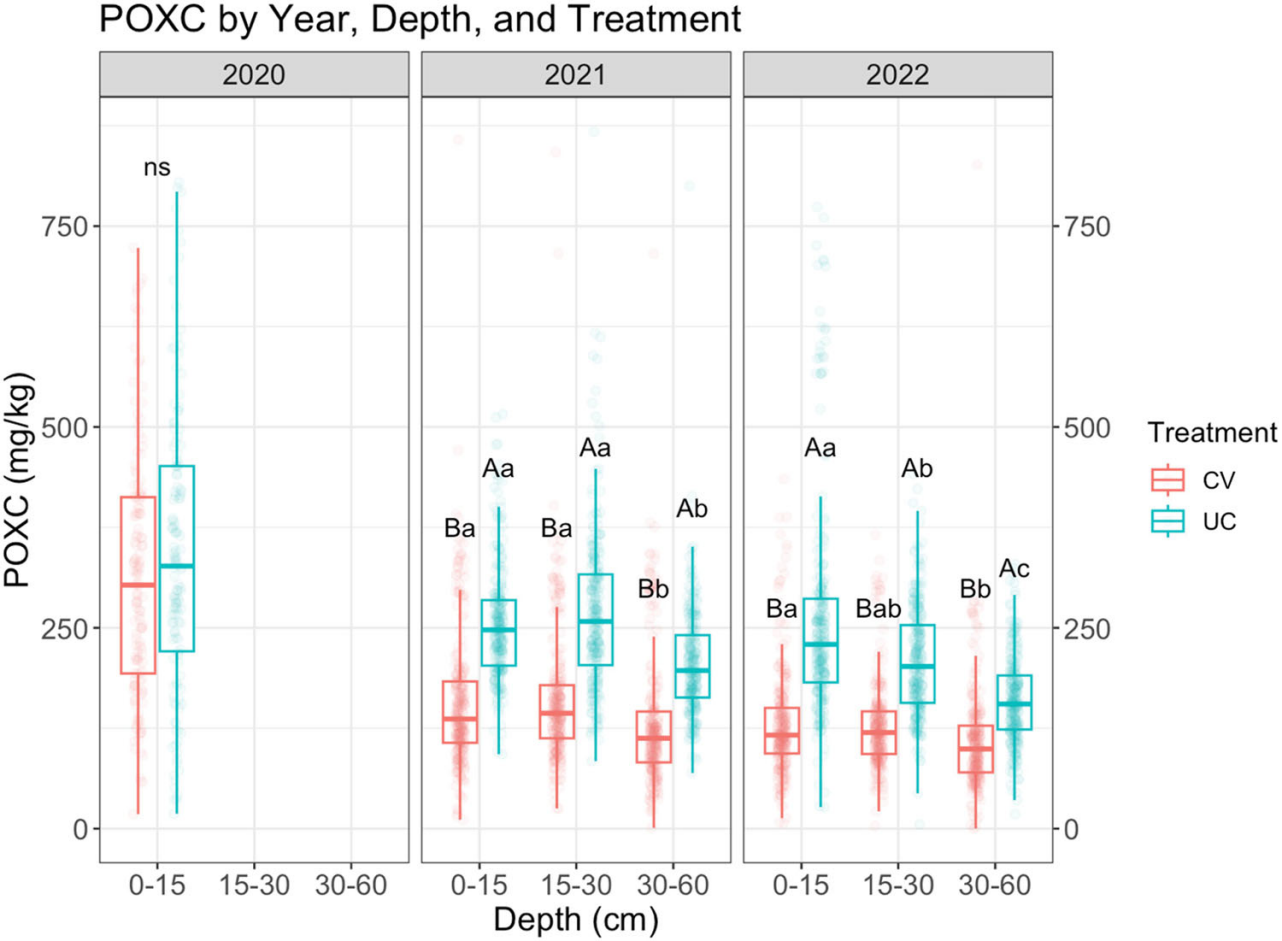
Boxplot of soil permanganate oxidizable carbon (POXC) content across different depths, treatments, and years. The graph is faceted by year, and different colors represent each treatment. Significant differences (*p* < 0.05) between treatments within the same depth and year are indicated by different upper‐case letters, while differences between depths within the same treatment and year are denoted by different lower‐case letters (ns: not significant). CV, covered; UC, uncovered.

In 2021 and 2022, samples were collected at three different depths (0–15, 15–30, and 30–60 cm). Table 2 summarizes the R output of the type III analysis of deviance. All the fixed effects included in the model were significant, as were their interactions (*p* < 0.001). Post hoc pairwise comparisons (Table 3; Figure 4) show the trend of average POXC change over time by year, depth, and treatment. In 2021, under the CV treatment, the average POXC was 159 mg kg^−1^ at a depth of 0–15 cm and 163 mg kg^−1^ at a depth of 15–30 cm (Figure 4; Table S2). Under the UC treatment, the average POXC was 256 mg kg^−1^ at depths of 0–15 cm and 269 mg kg^−1^ at depths of 15–30 cm (Figure 4; Table S2). No significant difference between the 0‐ to 15‐cm and 15‐ to 30‐cm depths could be detected for either treatment. However, for both treatments, at 30‐ to 60‐cm depth, we found significantly lower POXC compared to both 0‐ to 15‐cm and 15‐ to 30‐cm depths, with values of 132 and 207 mg kg^−1^ for CV and UC, respectively (Figure 4; Table S2). In 2022, under the UC treatment, all three depths were significantly different from each other (*p* < 0.05). Average POXC was 261 mg kg^−1^ at 0‐ to 15‐cm depth, 209 mg kg^−1^ at 15‐ to 30‐cm depth, and 159 mg kg^−1^ at 30‐ to 60‐cm depth (Figure 4; Table S2). Under the CV treatment, the average POXC content was 130 mg kg^−1^ at 0‐ to 15‐cm depth, 129 mg kg^−1^ at 15–30 cm, and 111 mg kg^−1^ at 30‐ to 60‐cm depth (Figure 4; Table S2). The difference in POXC content between 30‐ to 60‐cm depth and both the 0‐ to 15‐cm and 15‐ to 30‐cm depths was significant (*p* < 0.05) (Figure 4; Table S2). At 0‐ to 15‐cm depth, for which data from all 3 years are available, it is evident that POXC under the UC treatment remained stable between 2021 and 2022, whereas POXC under the CV treatment steadily decreased (Figure S5). Figure S5 shows the variation in POXC content for each of the depths between 2020 and 2022, making it easier to visualize the trends.

**TABLE 2:** Summary of the type III analysis of deviance for permanganate oxidizable carbon (POXC) carried out in R software.

Source	Wald *χ* ^2^	*df*	*p*‐value
Intercept	359.4	1	<0.001
Treatment	6.6	1	0.010
Year	327.3	2	<0.001
Depth	7.3	2	0.02
Treatment:Year	56.2	2	<0.001
Treatment:Depth	55.3	2	<0.001
Year:Depth	1.4	2	0.49
Treatment:Year:Depth	19.3	2	<0.001

*Note*: All of the fixed effects included in the model and their interactions are significant 0.05 significance level, except the interaction between year and depth.

Abbreviation: df, degrees of freedom.

**TABLE 3:** Post hoc pairwise comparisons carried out through the emmeans() function in the emmeans package in R software.

Treatment	Year	Contrast between depths (cm)	Difference in POXC estimate	SE	*Z* ratio	*p*‐value
CV	2021	0–15 vs. 15–30	−4.1	7.91	−0.520	0.8617
		0–15 vs. 30–60	27.1	7.96	3.399	**0.0020**
		15–30 vs. 30–60	31.19	7.93	3.935	**0.0002**
	2022	0–15 vs. 15–30	0.70	7.89	0.089	0.9956
		0–15 vs. 30–60	18.84	7.90	2.384	**0.0451**
		15–30 vs. 30–60	18.13	7.90	2.295	0.0565
UC	2021	0–15 vs. 15–30	−13.79	8.19	−1.684	0.2113
		0–15 vs. 30–60	48.65	8.20	5.934	**<0.0001**
		15–30 vs. 30–60	62.44	8.21	7.609	**<0.0001**
	2022	0–15 vs. 15–30	51.76	7.95	6.513	**<0.0001**
		0–15 vs. 30–60	101.43	7.95	12.761	**<0.0001**
		15–30 vs. 30–60	49.66	7.95	6.248	**<0.0001**

*Note*: *p*‐values were Tukey adjusted. *p*‐values in bold are considered significant. No significant difference in average permanganate oxidizable carbon (POXC) level was observed between 0‐ to 15‐cm and 15‐ to 30‐cm depths, except in the control treatment in 2022, where the average POXC level at 15‐ to 30‐cm depth was significantly lower than that at 0‐ to 15‐cm depth.

Abbreviations: CV, covered; SE, standard error; UC, uncovered.

**FIGURE 5:**
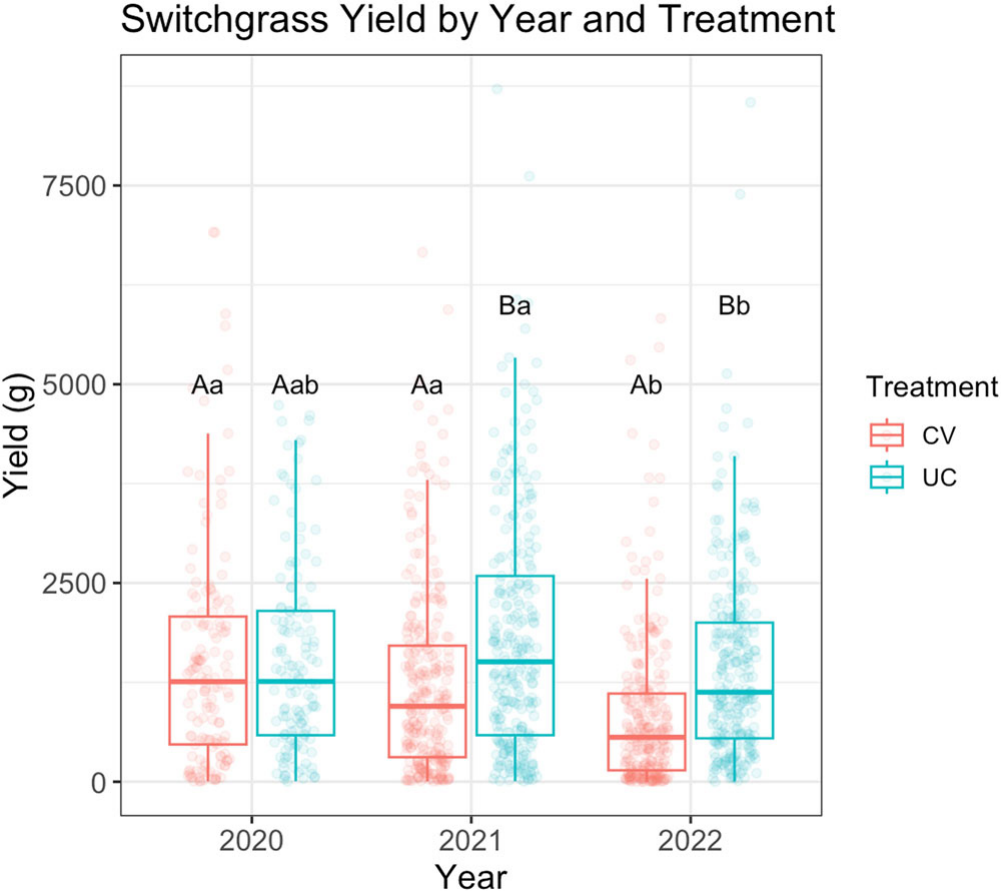
Boxplot of switchgrass yield by treatment and year. Different colors indicate different treatments. Yield in 2020 is comparable between treatments. In the following years, the average yield in the control (uncovered [UC]) is higher than the average yield in the plot under drought treatment (covered [CV]). Significant differences (*p* < 0.05) between treatments within the same year are indicated by different upper‐case letters, while differences between years within the same treatment are denoted by different lower‐case letters. CV, covered; UC, uncovered.

In 2020, there was no significant difference in biomass yield per plant between the treatments: average yield was 1.563 kg under the UC treatment and 1.511 kg under the CV treatment (Figure 5; Table S3). In the following years, the average yield in the UC plots was greater than the CV plots and higher than the 2020 average. In 2022, the average yield decreased under both treatments (Figure 5; Table S3). Table 4 summarizes the type III analysis of deviance. All the fixed effects included in the model were significant, as well as their interactions (*p* < 0.001). Post hoc pairwise comparisons are reported in Table 5.

**TABLE 4:** Summary of the type III analysis of deviance for yield carried out in R software.

Source	Wald *χ* ^2^	*df*	*p*‐value
Intercept	31.17	1	<0.001
Treatment	8.07	1	0.0044
Year	31.07	1	<0.001
Treatment:Year	8.08	1	0.0044

*Note*: All of the fixed effects included in the model and their interactions are significant at 0.05 significance level.

Abbreviation: df, degrees of freedom.

**TABLE 5:** Post hoc pairwise comparisons between treatments carried out through the emmeans() function in the emmeans package in R software.

Year	Treatment contrast	Estimate	SE	*df*	*t* ratio	*p*‐value
2020	CV vs. UC	−24.5	149	1347	−0.164	0.8695
2021	CV vs. UC	−582.8	107	1347	−5.466	**<0.0001**
2022	CV vs. UC	−613.3	107	1347	−5.728	**<0.0001**

*Note*: *p*‐values were Tukey adjusted. Degrees of freedom (df) are computed using the Kenward–Roger method. *p*‐values in bold are considered significant.

Abbreviations: CV, covered; SE, standard error; UC, uncovered.

### 3.4 Relationship between yield and POXC

**FIGURE 6:**
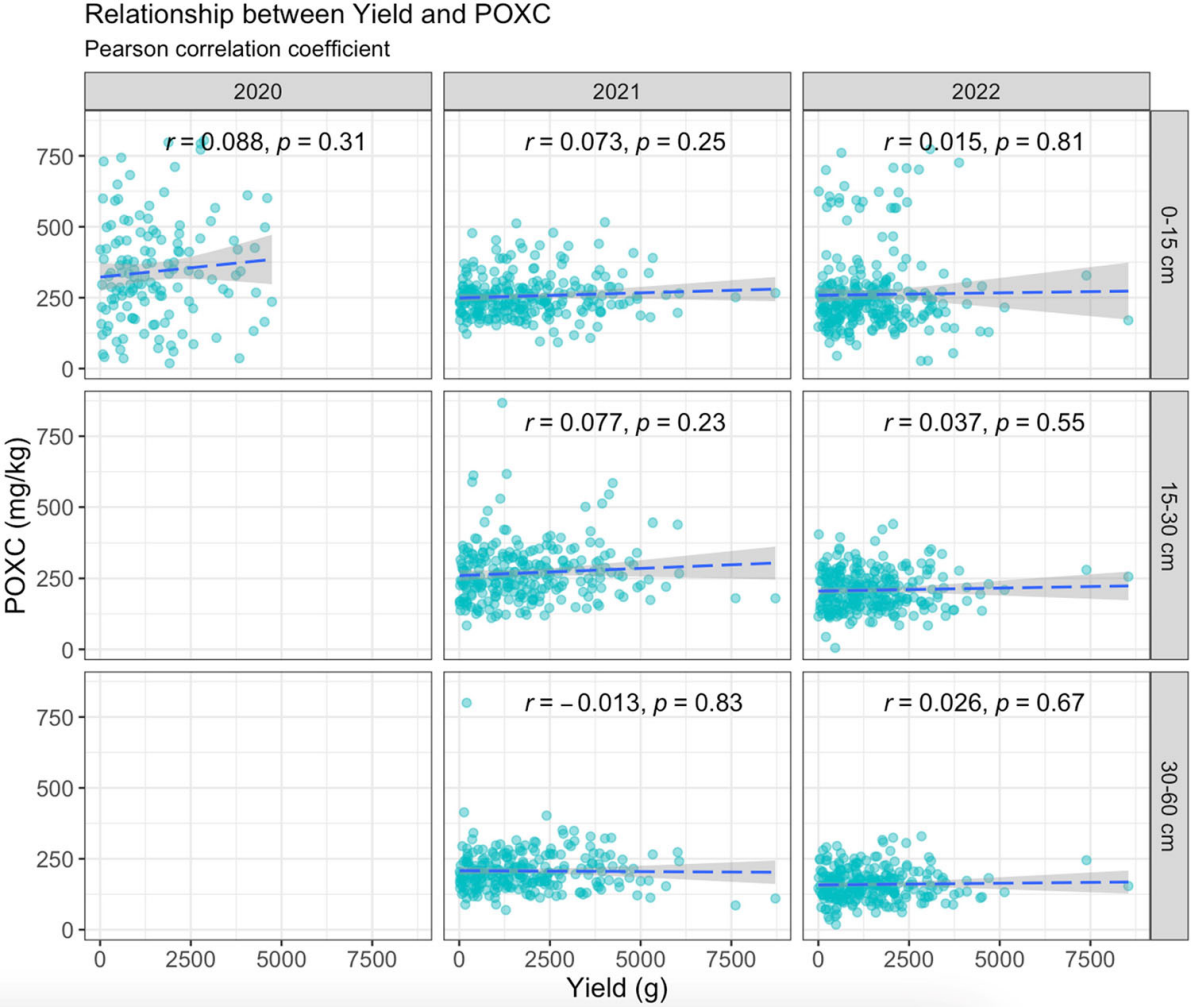
Correlation between yield and permanganate oxidizable carbon (POXC) for every combination of year and depth under study in uncovered (UC) (control) treatment. Depth 1 represents 0–15 cm, depth 2 represents 15–30 cm and depth 3 represents 30–60 cm. No significant correlation between yield and POXC was observed for any of the years and depths studied.

**FIGURE 7:**
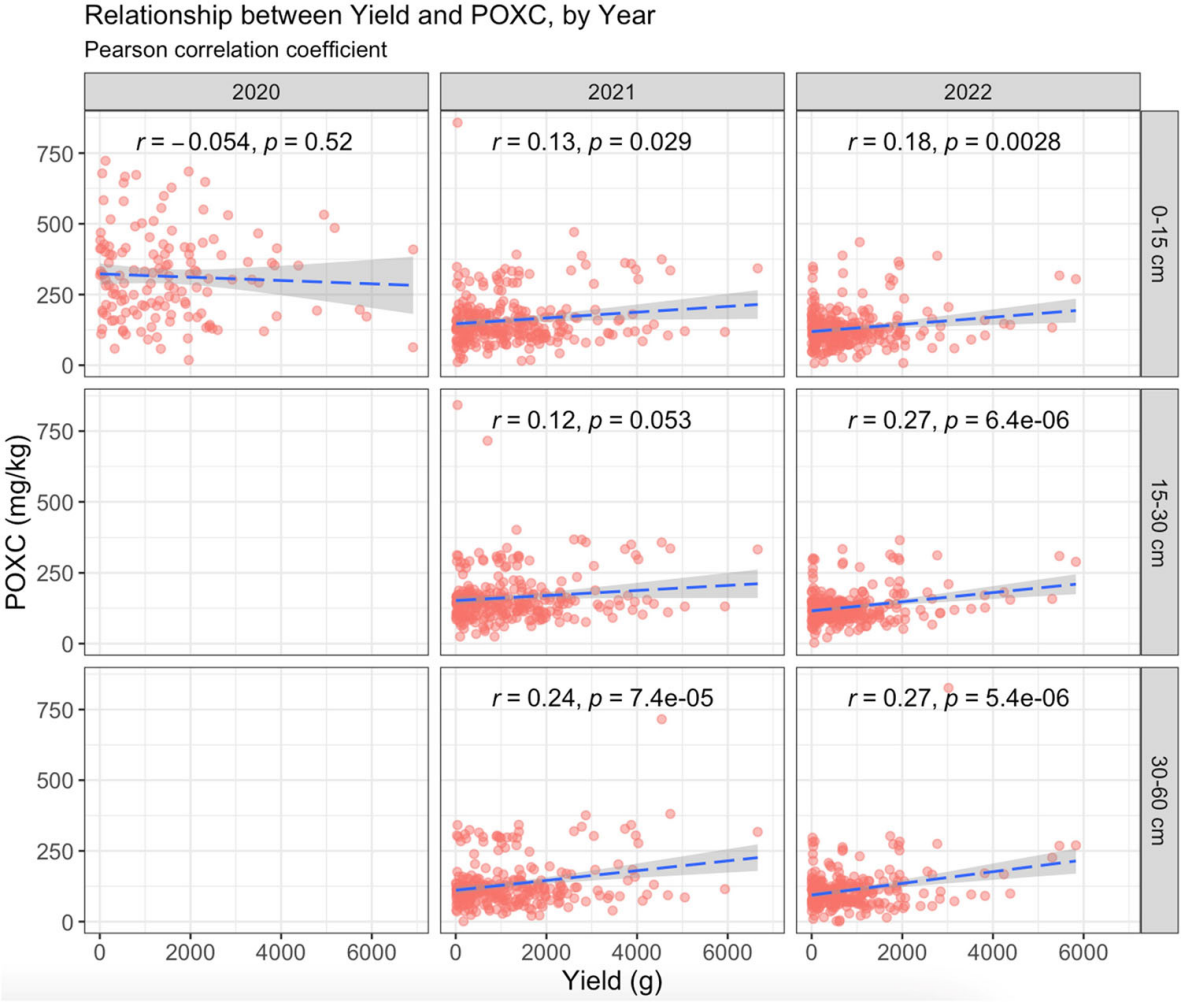
Correlation between yield and permanganate oxidizable carbon (POXC) for every combination of year and depth under study in covered (CV) (drought) treatment. Depth 1 represents 0–15 cm, depth 2 represents 15–30 cm and depth 3 represents 30–60 cm. A slightly positive correlation between yield and POXC becomes particularly evident and significant in 2022 as sampling depth increases.

Under the UC treatment, there was no correlation between yield and POXC for any of the years or depths studied (Figure 6). Under the CV treatment, a slightly positive correlation between yield and POXC was observed in both 2021 and 2022 (Figure 7). The correlation was significant at 0–15 cm (*r* = 0.13, *p* = 0.029) and 30–60 cm (0.24, *p* < 0.0001) depths in 2021 and at all depths in 2022 (0–15 cm: *r* = 0.18, *p* = 0.0028; 15–30 cm: *r* = 0.27, *p* < 0.0001; 30–60 cm: *r* = 0.27, *p* < 0.0001) (Figure 7). Pearson correlation and Spearman rank correlation coefficients for each combination of treatment, year, and depth of interest are summarized in Table 6.

**TABLE 6:** Pearson and Spearman rank correlation coefficients between yield and permanganate oxidizable carbon (POXC) for every combination of year, treatment, and depth of interest.

Depth (cm)	Pearson (2021)		Spearman (2021)		Pearson (2022)		Spearman (2022)	
	UC	CV	UC	CV	UC	CV	UC	CV
0–15	0.113	0.122	0.0820	0.048	0.018	0.181	0.047	0.038
15–30	0.084	0.107	0.1230	0.075	0.040	0.271	0.023	0.111
30–60	−0.007	0.223	0.0734	0.098	0.025	0.274	0.009	0.107

*Note*: Comparatively higher correlation values were observed under the covered (CV) treatment in 2022 at the two deeper sampling depths (15–30 cm and 30–60 cm).

Abbreviations: CV, covered; UC, uncovered.

## 4 DISCUSSION

POXC is an indicator of readily accessible carbon that meets microbial energy demands, or in other terms, microbial decomposition (Culman et al., 2012; Hurisso et al., 2016; Burke et al., 2019) and may help illustrate carbon dynamics in the plant‐soil continuum in our switchgrass system. Although total SOC does not respond quickly to the addition of switchgrass aboveground biomass residue as well as belowground root, the readily available soil carbon portion may show a rapid response (Ma et al., 2000a, 2000b). Switchgrass growth in the initial years is typically below full potential—about one‐third in the first year, two‐thirds in the second year, and full potential from the third year onward. In 2020, variability in growth and the minimal treatment effect may also reflect establishment effects and environmental fluctuations during the early stand phase. Differences between treatments in terms of yield became more distinct in subsequent years as stands matured and soil moisture differentials stabilized between the drought and control plots. The observed lack of significant differences in soil POXC content at baseline in 2020 between the two study sites indicates that the difference in previous management did not have any impact on the soil carbon pool measured by POXC (Figure 4). When looking at other soil characteristics, we saw overall lower levels of %TOC, %OM, pH, %BS, and %CEC in the CV plot compared to the UC plot. Drought stress, therefore, seemed to have a detrimental effect on soil quality in the CV plot. In 2021 and 2022, at all depths, significantly greater POXC in the UC treatment compared to CV was most likely due to the lack of available soil moisture in the root‐microbe interaction zone. In 2021, for both treatments, POXC was significantly lower in the deep soil (30‐ to 60‐cm depth) compared to the shallower depths, possibly due to the lack of root‐microbe interaction, thereby slowing microbial‐mediated processes at deeper depths (Stewart et al., 2017). The lower POXC content under the CV treatment starting in 2021 onwards is likely caused by the detrimental impact of water limitation on both switchgrass growth and SOC content (Deng et al., 2021). Overall, POXC continued to decrease in the covered section in 2022. In 2022, UC showed a significantly decreasing trend in POXC with depth (POXC at 0–15 cm > POXC at 15–30 cm > POXC at 30–60 cm). This trend of POXC decrease with increasing depth may indicate that readily available carbon or the input of labile carbon for microbial decomposition decreases with depth (Crowell et al., 2022; Frene et al., 2020). Furthermore, whether this trend is due to seasonal variation or represents a consistent phenotypic behavior remains to be investigated through additional sampling rounds in the long term. Under both treatments, POXC was significantly lower at the 30‐ to 60‐cm depth, possibly due to the combination of two factors: (i) lower switchgrass root biomass at deeper depths and (ii) lack of water in deeper soil layers, especially under drought treatment. In the drought treatment, we found a positive correlation between yield and POXC, particularly in 2022 and at greater soil depths. This suggests that the relationship is mainly detectable under drought conditions and tends to become more evident over time and with increasing depth. This observation is consistent with previous findings of Liebig et al. (2005).

The correlation coefficients (Table 6) further support this pattern. Under drought (CV) conditions, both Pearson's and Spearman's values showed a consistent, weak but significant positive relationship between yield and POXC, particularly in 2022 at the 15‐ to 30‐cm and 30‐ to 60‐cm depths (*r* = 0.27, *p* < 0.05). The agreement between the two correlation methods confirms that this trend was robust and not influenced by outliers. This suggests that genotypes maintaining higher yield under drought also maintained greater active carbon measured as POXC in both 15‐ to 30‐cm and 30‐ to 60‐cm depths, likely due to enhanced root biomass that potentially can induce microbial activity and further contribute to the storage of more stable carbon in deeper soil depths. In addition, drought stress resulted in a significant yield reduction compared to the control (Table 5; Figure 5). This substantial decline highlights the sensitivity of switchgrass productivity to water limitation and underscores the importance of developing genotypes with improved drought resilience and root traits that enhance soil carbon storage, particularly at deeper depths.

The positive correlation between POXC and yield observed under drought (Table 6) suggests that genotypes with greater biomass production may also have increased the root‐derived carbon in the soil. The belowground translocation of soil carbon and its incorporation into the soil microbial system and soil organic matter have been reported by Kuzyakov and Domanski (2000). Carbon flow in the rhizosphere is highly dynamic, with root‐derived carbon both released into the soil and reabsorbed by roots, varying with plant type and environmental conditions (Jones et al., 2009).

The drought response nature of genotypes can affect the correlation between POXC and yield. If more drought‐resistant genotypes had been included, the positive correlation observed under drought could have been stronger, due to the ability of such genotypes to maintain higher root growth, efficient photosynthetic activity, deeper rooting, and greater rhizodeposition even under drought (Kell, 2012; Kuzyakov & Domanski, 2000). Most soil carbon originates from plant photosynthesis and is transferred into the soil through the roots, with soils storing approximately twice as much carbon as the atmosphere, yet still having room to store more (Kell, 2012). Switchgrass genotypes vary in their ability to sustain biomass under drought conditions due to strong genotype‐by‐environment interactions. Makaju et al. (2025) developed a Drought Adaptation Index and identified genotypes with high and stable performance across drought and control conditions. Their analyses showed that drought resistance in switchgrass is highly genotype‐specific and depends on the growing environment.

In addition to plant productivity, several soil and rhizosphere processes may influence POXC. Root exudation, microbial decomposition, and organic matter turnover all contribute to the formation and stabilization of labile carbon pools (Kuzyakov & Domanski, 2000). The dynamic nature of carbon flow in the rhizosphere, where root‐derived carbon is both released and reabsorbed, can further regulate short‐term POXC levels depending on plant‐microbe interactions and environmental conditions (Jones et al., 2009). These mechanisms highlight that POXC reflects not only plant yield responses but also broader soil biological activity.

POXC has recently been used as an indicator of soil accessible carbon (Culman et al., 2012) to evaluate the impact of cropping systems and agricultural practices on soil health (Malone et al., 2024; Pokhrel et al., 2024; Thapa & Mowrer, 2024). This study provides a framework for evaluating switchgrass as a sustainable biofuel crop, which improves both soil health and soil carbon content through POXC measurements over time. If switchgrass becomes a relevant and sustainable source of biofuel feedstock, it will depend on the ability to develop and commercialize a cultivar that meets both economic and sustainability standards (Field et al., 2018). While economic standards are mainly influenced by yield, yield stability, and agronomic practices, sustainability standards depend on the resources necessary to cultivate switchgrass efficiently and on the overall carbon balance of switchgrass‐based biofuel production. This study investigated the potential of switchgrass to sequester carbon in the soil and the impact of water limitation on this process.

While this study provides valuable insights into the relationship between water limitation, POXC, and yield, several limitations should be acknowledged. First, the experiment was conducted at a single site with specific soil and climatic conditions, which may restrict the generalization of results to other agroecoregions. Second, the study duration reflects short‐term responses to drought but may not capture longer‐term soil carbon dynamics under continued management. Third, while the active fraction of soil carbon measured as POXC often indicates to short‐term management changes (Dahal et al., 2020), due to the limited years of the study, the more stable pool/recalcitrant pool of carbon that is strongly associated with carbon sequestration by root decay at different rooting depths could not be measured.  Fourth, POXC was used as a proxy for labile soil carbon. Integrating multiple aspects, such as the drought response nature of genotypes, the soil microbial system, inclusion of multiple sites, long‐term evaluation, and other POXC‐associated traits, would strengthen the predictive capacity of POXC as an indicator of soil carbon resilience in bioenergy systems. Future studies should explore the long‐term trend of soil carbon under switchgrass and the effect of soil physical and chemical characteristics, as well as perform a technical, economic, and life cycle analysis to account for the value of carbon fixed in the soil within the feedstock production system. Further monitoring of the effect of water limitation on POXC content through repeated measurements over time and soil microbiome analysis should be investigated.

## AUTHOR CONTRIBUTIONS

**Anita Giabardo**: Data curation; formal analysis; investigation; methodology; project administration; visualization; writing—original draft; writing—review and editing. **Kishan Mahmud**: Conceptualization; data curation; formal analysis; investigation; methodology; resources; supervision; writing—review and editing. **Shiva Makaju**: Data curation; formal analysis; investigation; methodology; resources; writing—review and editing. **Gary Hawkins**: Conceptualization; methodology; writing—review and editing. **Ali Missaoui**: Conceptualization; funding acquisition; project administration; resources; supervision; writing—review and editing.

## CONFLICT OF INTEREST STATEMENT

The authors declare no conflicts of interest.

## Supporting information



Supplemental materials include layouts of the experimental plots (Fig. S1 and Fig. S2), a picture of the soil sampling setup (Fig. S3), a boxplot showing soil POXC at baseline (2020) (S4), line plots showing the average POXC values at all depths, treatments, and years (Fig. S5), a table with average climatic data in Tifton GA (Table S1), and tables with mean, standard deviation and standard error for each combination of treatment and year for yield (Table S1) and POXC (Table S2).
