# Supplementary material for: Variation in carbon sequestration in response to water limitation in a diverse panel of switchgrass genotypes
Source: J Environ Qual. 2025 Dec 11;55(1):e70118. doi: 10.1002/jeq2.70118 (PMC12698906; doi:10.1002/jeq2.70118)
Supplement: Supplementary file 1 — Supplemental materials include layouts of the experimental plots (Fig. S1 and Fig. S2), a picture of the soil sampling setup (Fig. S3), a boxplot showing soil POXC at baseline (2020) (S4), line plots showing the average POXC values at all depths, treatments, and years (Fig. S5), a table with average climatic data in Tifton GA (Table S1), and tables with mean, standard deviation and standard error for each combination of treatment and year for yield (Table S1) and POXC (Table S2). [file JEQ2-55-0-s001.docx]

**Supplemental Material**


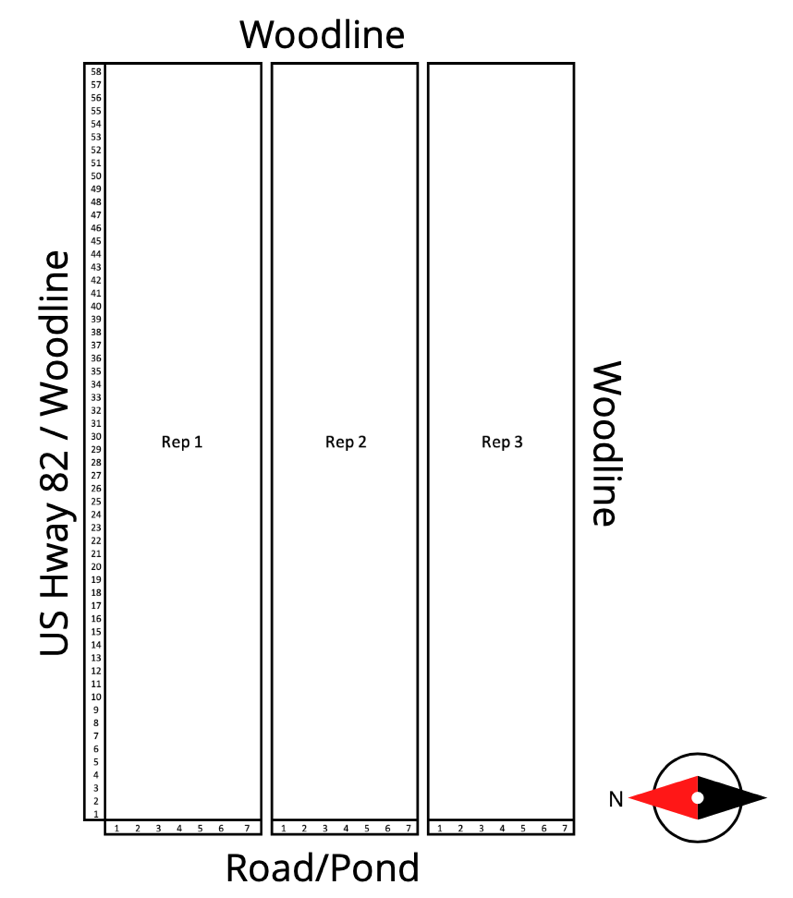


Figure S1. Layout of the CV (drought treatment) plot. The three replicates each contain 7 rows with 58 ranges per row. Within each replicate, the accessions are randomly arranged to control for bias in placement and spatial variation.


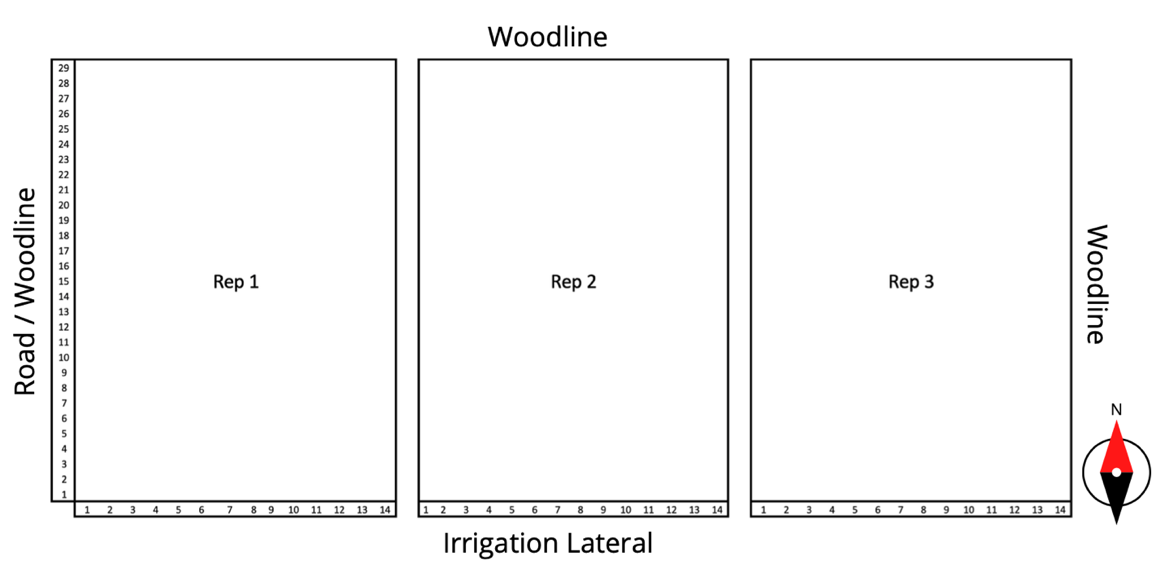


Fig. S2. Layout of the UC ("uncovered", or control) plot. The three replicates each contain 14 rows with 29 ranges per row. Within each replicate, the accessions are randomly arranged to control for bias in placement and spatial variation.


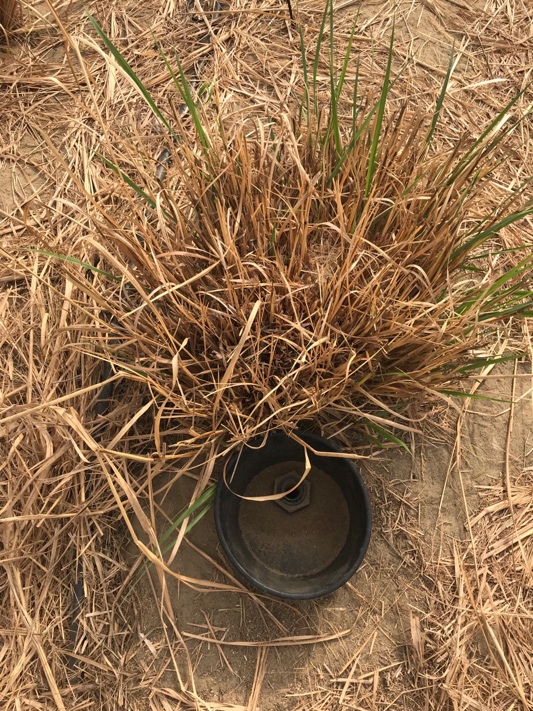


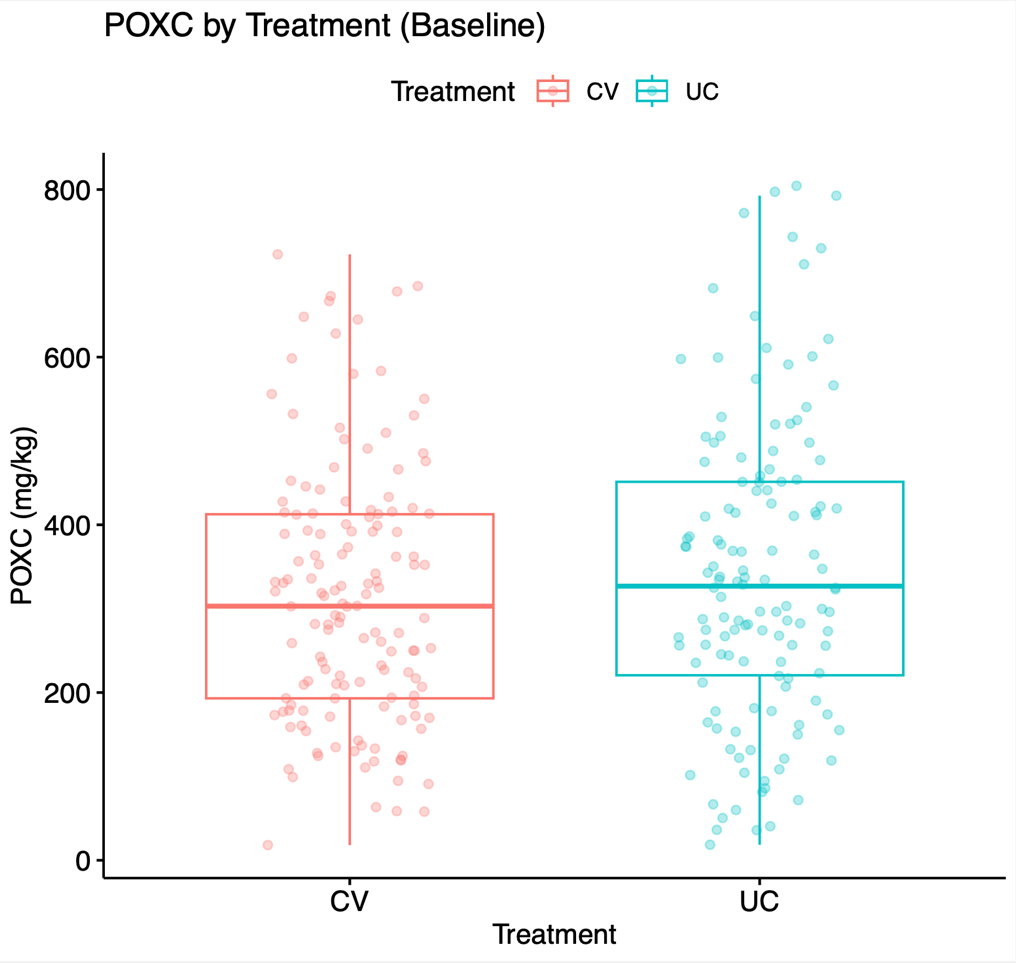
Fig. S3. Soil collection bucket with a hole for drilling. The hole is located near the edge of the bucket, to allow for sampling closer (about 5 cm) to the plant crown.

Figure S4. Boxplot showing soil POXC content (mg/kg) at baseline (2020), by treatment. Different colors indicate different treatments. Each data point represents one individual sample. All samples were taken from replication 2, at 0-15 cm depth. There is no statistically significant difference in the average POXC level of the two plots (F_1, 274_ = 1.9, p-value = 0.16).


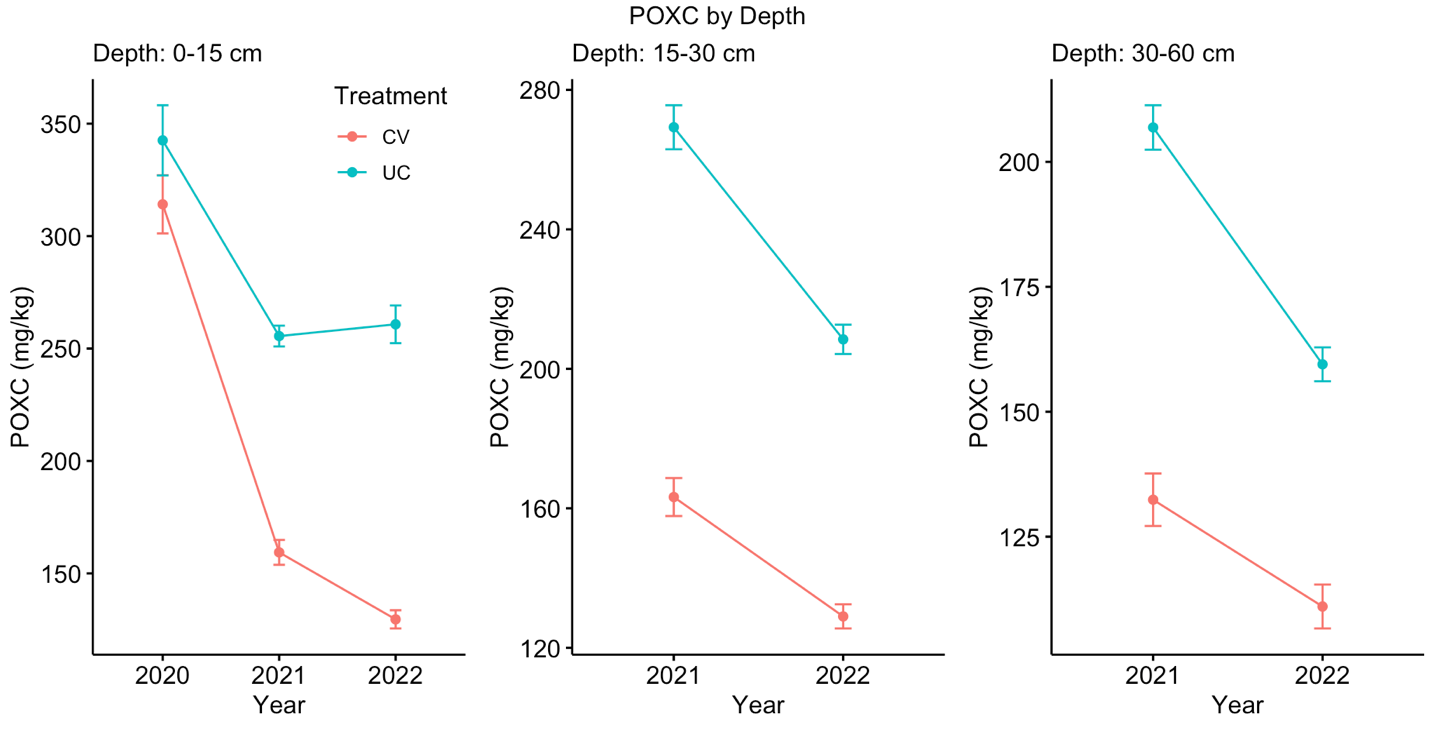


Figure S5. Average POXC values (± SE) at 0-15 cm (left), 15-30 cm (middle), and 30-60 cm (right) depths for different treatments across years. Different colors represent different treatments. The figures illustrate the divergence in POXC over time depending on the treatment. At the 0-15 cm depth, under both treatments, average POXC tends to decrease in 2021. In 2022, POXC remains stable for the UC (control) treatment, while it further decreases for the CV (drought) treatment. At both the 15-30 cm and 30-60 cm depths, under both treatments, average POXC tends to decrease in 2022.

Table S1. Average climatic data in Tifton, GA (31.446 N, 83.477 W) from 1923 to 2016. Elevation: 116 m (UGA Weather Network, 2023).

| **Time Period** | **Average Maximum Temperature (°C)** | **Average Minimum Temperature (°C)** | **Total Precipitation (mm)** | **Number of Rainy Days** |
| --- | --- | --- | --- | --- |
| January | 16.0 | 3.6 | 108.458 | 9 |
| February | 17.6 | 4.8 | 106.68 | 9 |
| March | 21.2 | 8.2 | 122.174 | 9 |
| April | 25.4 | 12.1 | 98.806 | 7 |
| May | 29.2 | 16.5 | 82.296 | 8 |
| June | 32.0 | 20.2 | 117.094 | 11 |
| July | 32.7 | 21.5 | 137.922 | 14 |
| August | 32.7 | 21.3 | 123.952 | 13 |
| September | 30.7 | 19.0 | 96.774 | 9 |
| October | 26.3 | 13.0 | 57.658 | 6 |
| November | 21.2 | 7.7 | 63.5 | 7 |
| December | 17.0 | 4.5 | 92.456 | 9 |
| Year | 25.2 | 12.7 | 1,207.77 | 109 |

Table S2. Mean, standard deviation (sd), number of observations (n) and standard error of soil POXC for each combination of year, treatment, and depth.

| **Year** | **Treatment** | **Depth** | **Mean** | **sd** | **n** | **se** |
| --- | --- | --- | --- | --- | --- | --- |
| 2020 | CV | 0-15 | 314.1 | 154.3 | 142 | 12.9 |
| 2020 | UC | 0-15 | 342.5 | 180.9 | 134 | 15.6 |
| 2021 | CV | 0-15 | 159.3 | 90.4 | 267 | 5.5 |
| 2021 | CV | 15-30 | 163.2 | 89.7 | 272 | 5.4 |
| 2021 | CV | 30-60 | 132.3 | 85.4 | 265 | 5.2 |
| 2021 | UC | 0-15 | 255.5 | 73.4 | 267 | 4.4 |
| 2021 | UC | 15-30 | 269.2 | 99.9 | 266 | 6.1 |
| 2021 | UC | 30-60 | 206.8 | 70.2 | 265 | 4.3 |
| 2022 | CV | 0-15 | 129.7 | 66.7 | 271 | 4.0 |
| 2022 | CV | 15-30 | 128.9 | 56.8 | 271 | 3.4 |
| 2022 | CV | 30-60 | 110.8 | 71.9 | 269 | 4.3 |
| 2022 | UC | 0-15 | 260.6 | 136.0 | 268 | 8.3 |
| 2022 | UC | 15-30 | 208.8 | 68.7 | 268 | 4.2 |
| 2022 | UC | 30-60 | 159.1 | 55.1 | 268 | 3.3 |

Table S3. Mean, standard deviation (sd), number of observations (n) and standard error of switchgrass aboveground yield yield for each combination of year and treatment.

| **Year** | **Treatment** | **Mean** | **sd** | **n** | **se** |
| --- | --- | --- | --- | --- | --- |
| 2020 | CV | 1511.9 | 1419.3 | 142 | 119.1 |
| 2020 | UC | 1563.4 | 1214.3 | 134 | 104.9 |
| 2021 | CV | 1231.4 | 1180.2 | 272 | 71.5 |
| 2021 | UC | 1814.1 | 1493.8 | 269 | 91.0 |
| 2022 | CV | 825.0 | 951.1 | 272 | 57.6 |
| 2022 | UC | 1438.2 | 1184.9 | 268 | 72.3 |
